# APC^+/−^ alters colonic fibroblast proteome in FAP

**DOI:** 10.18632/oncotarget.241

**Published:** 2011-03-15

**Authors:** Bhavinkumar B. Patel, Xin-Ming Li, Maketa P. Dixon, Elena L. Blagoi, Emmanuelle Nicolas, Steven H. Seeholzer, David Cheng, Yin A. He, Renata A. Coudry, Sharon D. Howard, Dawn M. Riddle, Harry S. Cooper, Bruce M. Boman, Peggy Conrad, James A. Crowell, Alfonso Bellacosa, Alfred Knudson, Anthony T. Yeung, Levy Kopelovich

**Affiliations:** ^1^ Developmental Therapeutics, Fox Chase Cancer Center, Philadelphia, Pennsylvania; ^2^ Cancer Prevention and Control, Fox Chase Cancer Center, Philadelphia, Pennsylvania; ^3^ Cancer Biology, Fox Chase Cancer Center, Philadelphia, Pennsylvania; ^4^ Cell Culture facility, Fox Chase Cancer Center, Philadelphia, Pennsylvania; ^5^ Division of Genetic and Preventive Medicine, Thomas Jefferson University, Philadelphia, Pennsylvania; ^6^ University of California at San Francisco, San Francisco, California; ^7^ Division of Cancer Prevention, National Cancer Institute, Bethesda, Maryland

**Keywords:** Adenomatous polyposis, APC, tumor suppressor, colon fibroblasts, heterozygosity, proteomics

## Abstract

Here we compared the proteomes of primary fibroblast cultures derived from morphologically normal colonic mucosa of familial adenomatous polyposis (FAP) patients with those obtained from unaffected controls. The expression signature of about 19% of total fibroblast proteins separates FAP mutation carriers from unaffected controls (P < 0.01). More than 4,000 protein spots were quantified by 2D PAGE analysis, identifying 368 non-redundant proteins and 400 of their isoforms. Specifically, all three classes of cytoskeletal filaments and their regulatory proteins were altered as were oxidative stress response proteins. Given that FAP fibroblasts showed heightened sensitivity to transformation by KiMSV and SV40 including elevated levels of the p53 protein, events controlled in large measure by the Ras suppressor protein-1 (RSU-1) and oncogenic DJ-1, here we show decreased RSU1 and augmented DJ-1 expression in both fibroblasts and crypt-derived epithelial cells from morphologically normal colonic mucosa of FAP gene-carriers. The results indicate that heterozygosity for a mutant *APC* tumor suppressor gene alters the proteomes of both colon-derived normal fibroblasts in a gene-specific manner, consistent with a “one-hit” effect.

## INTRODUCTION

Abnormal gene expression changes in phenotypically normal, cultured epithelial and stromal cells have been demonstrated in a number of autosomal dominant cancer syndromes, including the Li-Fraumeni syndrome (*LFS*) [1], *BRCA1* and *2*, [2], the von Hippel-Lindau (*VHL*) and the tuberous sclerosis complex (*TSC*) [3] and familial adenomatous polyposis (FAP) patients [4]. For example, FAP mutation carriers are heterozygous for mutations in the adenomatous polyposis coli (*APC*) tumor suppressor gene, and wherein the proteome of epithelial cells within colonic crypts of normal-appearing colonic mucosa differs from that of control individuals with the wild-type *APC* gene [4].

Although colon cancer arises from crypt epithelial cells, one element of growth regulation of the epithelial cells *in situ* is provided by stromal fibroblasts which form a sheath around the colonic crypts. In fact, a tight temporal relationship was shown to exist between epithelial and stromal cells in the colon epithelio-mesenchymal matrix during cell differentiation [5]. Here we showed that the proteome of fibroblasts within crypts of normal-appearing colonic mucosa of FAP patients differs from wild-type *APC* gene carriers and that these alterations are shared by the colonic crypt epithelial cells, consistent with a role for mesenchymal-epithelial interaction and possibly a harbinger of epithelial mesenchyme transition (EMT) [5, 6]. These studies should provide more precise molecular markers likely unique for *APC* alterations which would enable mechanism-based early detection and personalized prevention strategies for colon cancer.

## RESULTS

### Characterization of FAP *versus* controls

**Figure 1:**
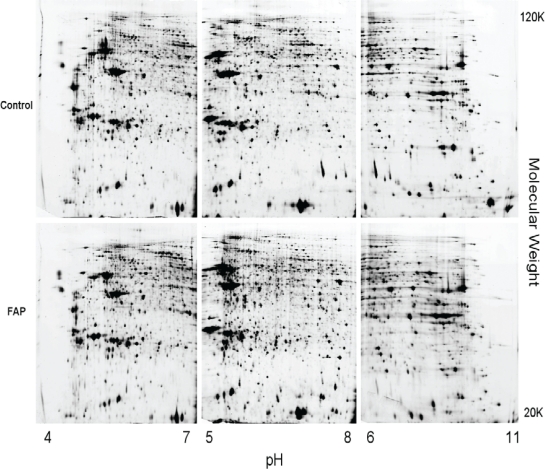
2D gels of FAP and control colonic fibroblasts.Three overlapping pH range 2D gels across p*I* 4—11 of FAP and control colonic fibroblasts These files are also in the Supplemental Data and at our web site after publication and are searchable and hyperlink-enabled to gene ontology on line.

Supplemental Data 1 tabulates characteristics of patient samples used for the study. Representative 2D gels of FAP and control colonic fibroblasts are shown in Figure 1. Both control and FAP samples were initially used to construct a protein and isoform database for the colonic fibroblasts (Supplemental Data 2—4), including mass spectrometry identification of about 368 unique protein entries and about 400 of their post-translationally modified isoforms from 2D gels. Each spot is hyperlinked to a gene ontology web site. Size and isoelectric point information allowed each spot to be scrutinized for post-translational modification. Point-and-click searchable proteomes for the colonic fibroblasts for three pH ranges are provided in supplemental data and at our web site. Proteins with expression changes are indicated in Supplemental Data 5—7 and tabulated in Supplemental Data 8. Many of the observed protein changes affect pathways of energy metabolism, cytoskeleton, proteolysis, protein synthesis, or RNA metabolism (Supplemental Data 8). Significant FAP-specific proteomic changes in common between crypt-derived epithelial cells and colonic fibroblasts are highlighted in Table 1.

**Table 1:** FAP-dependent protein differences found in both crypt epithelial cells & colonic fibroblasts

Colonic Fibroblast_FAP fold change, control = 1	Colonic Crypt_FAP fold change, control = 1	ID	2D gel pH range	Swiss-Prot ID #	Protein Name	Theoretical pI	Theoretical MW
FAP >2X Control							
2.8	4.8	2557	5-8	Q99497	Protein DJ-1	6.3	19891
Only in FAP	4.4	2097	4-7	P07339	Cathepsin D	5.6	37852
2.0	4.1	1553	5-8	P61163	α-centractin (Centractin)	6.2	42614
Only in FAP	3.2	2165	4-7	P52565	Rho GDP-dissociation inhibitor 1	5.0	23207
10.5/2.6	2.9	1390	5-8	P06733	α-enolase	7.0	47038
Only in FAP	2.8	1126	5-8	P30101	Protein disulfide-isomerase A3	5.6	54265
2.9	2.6	1892	4-7	P10768	Esterase D	6.5	31463
2.6	2.1	2454	5-8	P30041	Peroxiredoxin-6	6.0	24904
Control >2X FAP							
−2.1	−2.1	2448	5-8	P60174	Triosephosphate isomerase	6.5	26538
−3.3	−2.0	2114	5-8	P08758	Annexin A5	4.9	35806

The cytoskeleton is comprised of three types of filaments, each was observed to change in a gene-specific way in the FAP fibroblasts. These are the actin filaments, the intermediate filaments, and the microtubules (Table 2 and Supplemental Data 9).

**Table 2:** Differential expression between FAP and normal fibroblast primary cultures. Cytoskeleton proteins organized according to functional categories.

FAP fold change from Progenesis, control=1	pH range specific Unique No.	2D gel pH range	Swiss-Prot Accession No.	Protein Name	Theoretical pI	Theoretical MW	MASCOT Score	Amino Acid Coverage (%)
Cytoskeleton proteins organized according to functional categories								
Actin Filaments								
FAP Only	1669	4-7	P60709	Actin, cytoplasmic 1 (Beta-actin)	5.3	41606	60	22
−3.3	1049	5-8	P60709	Actin, cytoplasmic 1 (Beta-actin)	5.3	41606	89	32
FAP Only	2232	4-7	P62736	Actin, aortic smooth muscle	5.2	41775	57	29
−2.4	1048	5-8	P63267	Actin, gamma-enteric smooth muscle	5.3	41643	81	24
−2.5	1578	5-8	O15144	Actin-related protein 2/3 complex subunit 2	6.8	34333	77	35
FAP Only	894	4-7	P21333	Filamin A (Alpha-filamin)	5.7	280630	67	10
−2	1897	4-7	P52907	F-actin capping protein alpha-1 subunit	5.5	32792	62	37
2.3	1819	4-7	P09493	Tropomyosin 1 alpha chain	4.7	32709	198	54
FAP Only	1736	4-7	P09493	Tropomyosin 1 alpha chain	4.7	32709	113	38
2	1448	4-7	P61163	Alpha-centractin (Centractin)	6.2	42614	88	34
4	532	4-7	Q05682	Caldesmon (CDM)	5.6	93250	101	29
FAP Only	750	4-7	Q05682	Caldesmon (CDM)	5.6	93250	86	25
−5.2	1310	5-8	Q05682	Caldesmon (CDM)	5.6	93250	53	14
9.1	1755	4-7	Q14847	LIM and SH3 domain protein 1 (LASP-1)	6.6	29717	95	44
FAP Only	1815	4-7	Q14847	LIM and SH3 domain protein 1 (LASP-1)	6.6	29717	66	37
−6.3	901	5-8	Q9NVA2	Septin 11	6.4	49267	73	23
2.2	1690	4-7	Q15019	Septin-2 (NEDD5 protein homolog)	6.2	41487	75	31
Intermediate Filaments								
2.1	870	4-7	P02545	Lamin A/C (70 kDa lamin)	6.6	74139	211	43
FAP Only	889	4-7	P02545	Lamin A/C (70 kDa lamin)	6.6	74139	222	42
−3.2	658	5-8	P02545	Lamin A/C (70 kDa lamin)	6.6	74139	148	31
2.6	661	4-7	P20700	Lamin B1	5.1	66277	129	30
3.4	1430	4-7	P08670	Vimentin	5.1	53520	219	55
−2.6	1081	4-7	P08670	Vimentin	5.1	53520	148	35
FAP Only	1199	4-7	P08670	Vimentin	5.1	53520	186	39
5.8	1191	4-7	P08729	Keratin, type II cytoskeletal 7	5.5	51287	211	45
Microtubules								
FAP Only	1082	4-7	P07437	Tubulin beta-2 chain	4.8	49671	68	21
3.1	1761	4-7	P07437	Tubulin beta-2 chain	4.8	49671	93	19
3	1763	4-7	Q9BQE3	Tubulin alpha-6 chain	5	49895	84	27
FAP Only	1744	4-7	Q9BQE3	Tubulin alpha-6 chain	5	49895	55	20
Proteins link Cytoskeleton and Plasma Membrane								
−3.8	396	4-7	P12814	Alpha-actinin 1	5.3	103058	109	21
6.2	429	4-7	O43707	Alpha-actinin 4	5.3	104854	82	18
2.9	1637	5-8	P04083	Annexin A1	6.6	38583	94	35
2.2	1690	4-7	P07355	Annexin A2	7.6	38473	232	53
−3.4	2085	4-7	P08758	Annexin A5	4.9	35806	139	52
−2.6	561	4-7	P15311	Ezrin (p81)	6	69268	150	36
2.4	1321	4-7	Q13418	Integrin-linked protein kinase 1	8.3	51419	75	23
2.3	653	4-7	P26038	Moesin	6.1	67689	200	45
−2.1	961	4-7	P26038	Moesin	6.1	67689	62	20
FAP Only	222	4-7	P18206	Vinculin (Metavinculin)	5.5	123668	125	22
2.1	510	4-7	Q15942	Zyxin (Zyxin 2)	6.2	61277	60	23
FAP Only	2430	4-7	P52565	Rho GDP-dissociation inhibitor 1	5	23076	78	38
FAP Only	1124	4-7	P78371	T-complex protein 1, beta subunit	6	57357	66	18
2.7	2207	5-8	Q01995	Transgelin	8.9	22480	92	40
−5.4	2205	5-8	Q01995	Transgelin	8.9	22480	95	42

Actins: Several actin-associated proteins showed isoform expression changes (Table 2). Actins with isoforms changing significantly include beta actin and smooth muscle actin, suggesting that these fibroblasts are myofibroblasts. Myofibroblasts are implicated in the structure and function of colonic crypt [7]. Actin proteins, include filamin-A, cross-links actin filaments and can affect their structure, strength, and cell proliferation [8]. The Septins [9], a family of cytoskeletal GTPases, may participate in a protein complex regulating the structure and function, elongation, bundling or disruption of the actin cytoskeleton and microtubules. Similarly, LASP-1 is an important regulatory protein in the actin cytoskeleton interacting through the palladin isoforms [10]. Importantly, the FAP gene-specific changes in actin filaments activity, and cell proliferation is highlighted by alterations in three isoforms of caldesmon (Table 2). Caldesmon regulates actin-tropomyosin activated myosin MgATPase via inhibition of Ca^++^-dependent smooth muscle contraction [11].

The intermediate filaments: The lamin A/C and B1 isoform changes can regulate the structure and stability of the inner nuclear membrane. The vimentin intermediate filaments provide mechanical strength to fibroblasts and indirectly regulate cell adhesion. Vimentin dynamic rearrangement is regulated by dephosphorylation [12]. The vimentin changes observed in Table 2 are vimentin fragments; the native vimentin level did not appear to change. During apoptosis, vimentin is cleaved by multiple activated caspases, including caspase-3, -6 and —9, at distinct sites to yield proteolytic fragments [13]. Cleavage of vimentin also may facilitate nuclear condensation and subsequent fragmentation via disruption of the cytoskeletal network. The observed vimentin changes may be linked to the observed lamin changes in the FAP fibroblasts. Lastly, cytoskeletal keratin 7 was observed to up-regulate six fold in FAP fibroblasts (Table 2). How this keratin isoform induction affects the FAP fibroblasts is not yet clear, but its actions may be via cell motility and transformation to an epithelial or endothelial phenotype.

The microtubules: Both alpha and beta tubulin chains were observed to change in FAP (Table 2). These proteins function in a variety of cellular activities and are drug targets for cancer treatment [14]. Their role in the FAP cytoskeleton may be interactive with at least 15 isoforms of other proteins that link the cytoskeleton to the plasma membrane (listed at the bottom of Table 2).

Proteins linking the cytoskeleton to membrane dynamics up-regulated include Alpha-actinin 4, Annexin A1, Integrin-linked protein kinase 1, moesin, zyxin, vinculin, rho GDP-dissociation inhibitor 1, T-complex protein 1 β subunit, and transgelin (Table 2).

The changes in cytoskeleton proteome, including the induction of a Rho GDP-dissociation inhibitor 1 in FAP fibroblasts (Tables 1 & 2), and previous observation of Ras-dependent viral transformation sensitivity of FAP fibroblasts, focused our interest on a potential role for Ras activity which depends on membrane localization [15]. Consequently, we investigated the subcellular location and levels of the Ras suppressor protein RSU1 in the colonic fibroblasts. The confocal immunofluorescence image in Supplemental Data 10 indicated that RSU1 antigen is largely associated with the plasma membranes in FAP fibroblasts whereas most of the APC protein appeared nuclear. Collectively our proteomic data illustrate profound cytoskeletal changes in FAP fibroblasts in a gene-specific manner.

### Measurement of RSU1 expression

**Figure 2:**
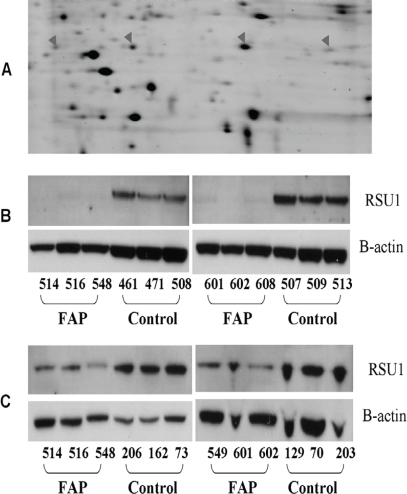
RSU-1 expression in colonic fibroblasts A. pH 6—11 range 2D gel analysis of RSU-1 protein isoforms in control colon fibroblasts. All the protein spots observed in this picture were identified with mass spectrometry. Four isoforms of RSU-1 are indicated by the arrows. The rightmost spot is unmodified RSU-1. The second spot from the left is lower in intensity in FAP fibroblasts. The right two RSU-1 spots were outside of the range of the pH 5—8 gels previously used to detect changes in RSU-1 levels. B. Western analysis of RSU1 expression in FAP colonic fibroblasts *versus* control colonic fibroblasts. C. Western analysis of RSU-1 expression in FAP colonic crypt cells *versus* those from control patients.

Consistent with a potential increase in Ras activity [16] we focused on RSU-1 that is a prime regulator of ras [17]. Validation of this potentially important biomarker, RSU1 was performed on morphologically normal crypt-derived epithelial cells and primary fibroblast cultures both from the morphologically normal mucosa of FAP gene carriers and unaffected controls (Figure 2). Spot 2474, identified as RSU1, averaged more than two-fold greater abundance in four control fibroblasts than in four FAP fibroblasts (Supplemental Data 11). The RSU1 spot, observed in the pH 5—8 gel, has the theoretical mass, yet its pI suggests that it may be a phosphorylated protein. Upon identification of all visible protein spots in this region of a pH 6—11 gel, four RSU1 isoforms of approximately the same size were identified (Figure 2A). These RSU1 spots in four 2D gels of FAP versus four gels of controls were quantified by densitometry scanning and the results are shown in Supplemental Data 12. The two RSU1 spots on the left side of the gel, significantly decreased in intensities in FAP cells (not detectable in FAP and 2.5 fold lower in FAP than control, respectively), show pI's consistent with modification by post-translational modifications, including but not limited to phosphorylation events.

RSU1 isoforms can also arise from alternate mRNA splice forms [18] and SNPs. The isoforms of different patients has slightly different electrophoretic mobilities (data not shown). Total RSU1 expression was lower in all six FAP fibroblast samples compared to non-FAP control fibroblasts (Figure 2B). RSU1 levels were also lower in the morphologically normal epithelial cell from FAP crypts than in control crypts (Figure 2C). It is also likely that RSU1 protein has more than one function in the cell, and a subset of this protein is modified in the FAP phenotype, wherein the lower level has potential to enhance the relative activity of the endogenous Ras oncogene.

### Oxidative Stress Response in FAP fibroblasts

The FAP fibroblasts exhibit an enhanced oxidative stress response (Table 3). Examples include elevation of Alpha crystallin B chain which is a heat shock protein that accumulates after oxidative stress conferring resistance to TNF and oxidative stress in murine fibroblasts [19], Other examples include *Tropomyosin*-1 which is phosphorylated at Ser283 through the Erk/DAPK pathway and promotes stress fiber formation in response to oxidative stress [20], and DJ-1. Yet another FAP-elevated protein is Peroxiredoxin-6, a phospholipase which protects mitochondria from ischemia and oxidative stress [21], by acting as the substrate for hyperoxidation [22]. Both DJ-1 and peroxiredoxin-6 are elevated in FAP colonic fibroblast cultures and in FAP crypt epithelial cells (Table 1 [4]).

**Table 3:** Differential expression between FAP and normal fibroblast primary cultures in response to oxidative stress. Both DJ-1 and Peroxiredoxin-6 were up-regulated in both FAP crypt and fibroblast.

FAP fold change from Progenesis, control=1	pH range specific Unique No.	2D gel pH range	Swiss-Prot Accession No.	Protein Name	Theoretical pI	Theoretical MW	MASCOT Score	Amino Acid Coverage (%)
Proteins involved in response to oxidative stress.								
FAP >2X Control								
4.1	2089	5-8	P02511	Alpha crystallin B chain (Rosenthal fiber component)	6.8	20159	138	46
2.8	2549	4-7	Q99497	DJ-1 protein (Oncogene DJ1)	6.3	19891	102	65
2.6	2388	4-7	P30041	Peroxiredoxin 6 (Antioxidant protein 2)	6.0	24904	100	42
2.3	1819	4-7	P09493	Tropomyosin 1 alpha chain (Alpha-tropomyosin)	4.7	32709	198	54
4.1	2089	5-8	P02511	Alpha crystallin B chain (Rosenthal fiber component)	6.8	20159	138	46
2.8	2549	4-7	Q99497	DJ-1 protein (Oncogene DJ1)	6.3	19891	102	65
Protein Found Only in FAP								
-	1736	4-7	P09493	Tropomyosin 1 alpha chain (Alpha-tropomyosin)	4.7	32709	113	38

### Measurement of DJ-1 expression

**Figure 3:**
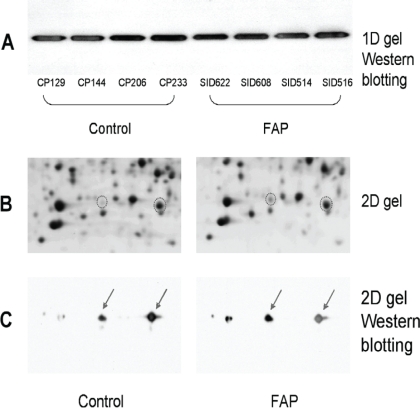
DJ-1 proteomic changes in colonic crypts A. 1D gel Western blotting, B. 2D gel pH4—7 12% SDS-PAGE gels, and mass spectrometry protein identification, and C. 2D gel Western blotting experiments. The circled protein spots were DJ-1 identified by mass spectrometry. Corresponding spots were indicated by arrows in the 2D Western blotting images. For the 2D Western, Control was CP 203, FAP was SID 516.

Our previous data on the potential role of p53 and oxidative stress [4] has also led us to focus on the role of another potential FAP biomarker, DJ-1. A DJ-1 isoform (Figure 3) identified as spot 2549 (in pH 4—7 gel) was up-regulated by 4.8-fold in FAP crypts-derived epithelial cells compared with controls (Supplemental Data 8) and up-regulated 2.8-fold in FAP colonic fibroblast cultures *versus* control cultures (Supplemental Data 13). Experiments using Western blots were performed to investigate DJ-1 expression changes in colonic crypts *versus* wild-type samples. Figure 3 illustrates the importance of identifying the protein spots that showed little change between cases and controls, and the need to follow through with 2D gel Western blot characterization. Not all isoforms of a given protein may show proteomic changes and some isoforms may be hidden by other co-migratory proteins. Alternatively, two isoforms of a protein may show reciprocal changes in expression. The level of DJ-1 did not change as judged by 1D gel Western blotting (Figure 3A). Although 2D gel protein identification by mass spectrometry only revealed two isoforms of DJ-1 (Figure 3B), 2D gel Western blotting experiment revealed at least four isoforms of DJ-1 (Figure 3C). The heaviest spot 1 on the right side represents the theoretical pI and molecular weight of the unmodified DJ-1 protein. Change in this spot was not detected in 2D gel images. However, spot 2 showed a pI value consistent with an increase of a post-translational modification, including but not limited to a phosphorylation event, in the protein molecule. This spot showed slight elevation in FAP samples. Spot 3 is the spot detected as up-regulated by about 3—4 fold in FAP crypt cells and in FAP fibroblasts. Spot 4 was also increased in FAP samples, but undetected in 2D gels by protein staining because it was hidden under a protein of greater abundance.

### Regulation of RSU1 and DJ-1 gene expression

Whether *RSU1* was regulated at the mRNA level in colonic fibroblasts was investigated by using quantitative real-time PCR. The transferrin receptor gene (*TFRC*) was used as the normalization gene. Results are shown in Supplemental Data 14. The fold protein levels of two RSU1 protein isoforms in FAP fibroblast ranged from not detectable in FAP to 2.5 fold lower in FAP than control, respectively (Supplemental Data 12B), and about 2.5 fold lower than control in Western blotting analysis (Figure 2B), and the mRNA level of *RSU1* was on average 1.15 ± 0.38 for control and 0.88 ± 0.15 for FAP relative to *TFRC*. This may suggests that expression of the *RSU1* protein is regulated primarily post-translationally. A similar pattern is seen with DJ-1, wherein the *DJ-1* mRNA level was statistically non-distinguishable by real-time PCR when six FAP fibroblast samples were compared with six controls (control mean Ct = 26.4, standard error = 0.325. FAP mean Ct = 26.3, standard error = 0.498) (Data not shown). In contrast, the protein level change of the DJ-1 isoform spot 3 was 3 to 4 fold greater in FAP fibroblasts.

## DISCUSSION

We recently reported proteome changes in crypt-derived epithelial cells isolated from normal appearing colonic mucosa of FAP patients *versus* control individuals [4]. About 13% of the protein spots in 2D gels were observed to shift in intensity and the changes resided mainly among isoforms of each protein [4]. Crypt epithelial cells are sheathed by and interactive with colonic fibroblasts [23]. The epithelial-mesenchymal matrix of the colon may play a major role during colorectal cancer progression, including the development of desmoid tumors that are prevalent in FAP patients, especially those with the Gardner variant [24]. To increase our understanding, we analyzed FAP-dependent protein changes in the colonic fibroblasts for systemic expression that may be linked to a FAP-specific phenotype.

Previously, we have demonstrated a disruption of overall actin organization in skin fibroblasts from FAP patients [25], including increased sensitivity to transformation by KiMSV and SV-40, and increased expression of the p53 protein [26-28]. Accordingly, we have focused on two proteins in our present database that are mechanistically important to such transformation and potentially relevant to the FAP phenotype; the Ras suppressor protein (RSU1) and the DJ-1 oncogene.

RSU1, so named because its absence led to v-Ras transformation in mouse cells, is reported to enhance Erk-2 activation, inhibit Jun kinase activation [29], and inhibit anchorage-independent growth of MCF7 cells [30]. More recently, full-length 33 KDa RSU1, but not a 29 KDa truncated form, was shown to bind to the LIM5 domain of the adaptor protein PINCH1 and to participate in adhesion-related functions [31]. Moreover, the 29 KDa form is elevated in cancer cell lines where Ras is activated. When cells were transformed with activated Ras, the RSU1 association with ILK and PINCH decreased and cell migration increased [32]. ILK expression was reported to be greatly elevated in the abnormal crypts from polyps of FAP patients [33]. The relationship of RSU1-ILK-PINCH1 interaction in the crypts and the potential role of RSU1 in cell migration are yet to be established. Interestingly, the APC protein itself has been implicated in cell-cell adhesion

Ceramide, a pro-apoptotic agent, has been shown to induce RSU1, presumably by directly suppressing Ras expression [34]. Significantly, we showed that RSU1 expression was decreased in both FAP fibroblasts and crypt epithelial cells, more dramatically in the former than the latter. Obviously, the decrease in RSU1 levels would allow enhanced endogenous Ras activity. The higher endogenous Ras activity may decrease ceramide-induced apoptosis and explain, in part, the 100- to 1000-fold enhanced sensitivity of the FAP fibroblasts to transformation by KiMSV by allowing full expression of the v-Ki-ras oncogene [26]. Consistent with the anti-apoptotic theme is the congruent observation of an isoform of Rho GDP-dissociation inhibitor 1 in FAP fibroblasts only and its elevation by 3.2 fold in FAP crypt when compared with control (Table 1). Elevation in this protein was shown to provide cancer cells with resistance to drug-induced apoptosis [35].

The second protein we focused on, DJ-1, has an isoform that is elevated by about three fold in fibroblasts and about five fold in crypt epithelial cells in a FAP specific manner (Table 1). DJ-1 has multiple known functions (CAP1/RS/PARK7). It was originally cloned as a putative oncogene capable of transforming NIH-3T3 cells in cooperation with H-*ras*[36]. DJ-1 is an integral mitochondrial protein and is a redox-dependent molecular chaperone that inhibits α-synuclein aggregate formation [37] and in the unfolded protein response countering protein damage from cigarette smoke in the human lung [38]. Recent research also implicated DJ-1 as important in lung [39] and prostate cancers [40]. Increased DJ-1 was of particular interest to us because our previous proteomic study in FAP crypts revealed an elevation of oxidative stress response and attenuation of apoptosis [4]. This DJ-1 elevation, linked to oxidative stress, is corroborated by the elevation of protein disulfide-isomerase A3 and peroxiredoxin-6 (Table 1). Protein disulfide-isomerase A3, uniquely present in FAP fibroblasts, and about three fold elevated in FAP crypts compared with control (Table 1) is needed to renature proteins that have been damaged by oxidative stress. Peroxiredoxin-6 (antioxidant protein 2), 2.6 fold elevated in colonic fibroblasts and 2.1 fold elevated in colonic crypts, is an anti-oxidative protein with phospholipase A2 activity that can promote the invasiveness of lung cancer cells [41].

Importantly, DJ-1 appears to have both positive and negative effects on p53 transcription. The caspase-6-derived C-terminal fragment of DJ-1 has been proposed to account for DJ-1 associated p53-dependent cell death [42]. Sumoylation of DJ-1 was also reported to repress p53 transcription [43]. On the other hand, sumoylation of DJ-1 and p53 reactivates p53 transcription through a Topors/p53BPs complex [44]. These mechanisms might be responsible for the increased stability of the p53 protein in skin fibroblasts from FAP patients due to elevation of oxidative stress response as an early event during cancer progression [28]. Furthermore, the increased availability of p53 in FAP may explain, in part, the increased sensitivity of FAP cells to transformation by SV-40 [45] that is facilitated by direct interaction of p53 and large T-antigen [46].

Alterations in Ras and p53 appear critical during colorectal cancer development, and changes of RSU1 and DJ-1 shown here may precede and perhaps facilitate mutational activation associated with both Ras and p53 [47]. These alterations are shared by the colonic crypt epithelial cells and colonic fibroblasts, consistent with a role for mesenchymal-epithelial interaction and possibly a harbinger of epithelial mesenchyme transition [5, 6]. Importantly, the fibroblasts of a FAP patient were observed to undergo spontaneous immortalization [48].Thus, these alterations may well provide a further clarification of optimal targets at the “two-hit” stage of cancer development; those revealed in one-hit lesions would not include confounding secondary tumor effects. Collectively, they appear to be directly related to cancer and may be useful as biomarkers for early events in colorectal cancer development, leading to the identification of molecular targets and agents for cancer chemoprevention.

## MATERIALS AND METHODS

### Clinical specimens

Eleven FAP patients and 15 control subjects (Supplemental Data 1) were recruited with approval by the Institutional Review Boards of Fox Chase Cancer Center (protocol number: 00-852) and Thomas Jefferson University (protocol number: 00-0023). The 2D gel analysis included three FAP patients (mean age of 39.3 years, range 23 to 53 years, S.D. 8.8 years) (Supplemental Data 1). The controls were obtained with patient consent through pinch biopsy specimens of patients with no evidence of disease undergoing screening colonoscopy. Control patients for 2D gel analysis included three patients (mean age of 60 years, range 53 to 73 years, S.D. 6.5 years). Validation experiments used samples from six FAP patients and six control patients that were not used in the 2D gel proteomics analyses. FAP cases for validation included men and women previously diagnosed with FAP and whose corresponding *APC* gene mutations had been documented by DNA sequencing of circulating white blood cells (Supplemental Data 1). Because FAP patients often undergo colectomy at an early age, but screening for sporadic cancer starts at a later age, it was not possible to age-match FAP cases with controls.

### Preparation of primary colonic fibroblast cultures

Fibroblast cultures were prepared from colonic mucosa of normal appearance verified by a resident pathologist. The mucosal layer of colonic biopsy specimens was transported on ice in Ham's F-12 serum-free medium containing 100 U/ml penicillin, 100 μg/ml streptomycin, 10 μg/ml ciprofloxacin, 2.5 μg/ml gentamycin, 2.5 μg/ml amphotericin B, and 100 U/ml nystatin. Upon receipt, the tissue samples were finely minced, transferred to a 50 ml tube containing 15 ml of 0.2% collagenase (Sigma #C-0130) in the transport medium, and incubated for 1.5 hours at 37°C in a rotating water bath. Transport medium (15 ml) was added to the tube and the cells were collected by centrifugation at 1200 × g for 10 minutes. The cell pellet was washed four times for five minutes, each time with 25 ml of transport medium, and once with fibroblast culture medium (DMEM supplemented with 15% FBS, 4 mM L-glutamine, 110 mg/L pyruvate, 100 U/ml penicillin, 100 μg/ml streptomycin and 10 μg/ml ciprofloxacin). The cells were then placed into a T-12.5 cm^2^ flask pre-coated with fetal bovine serum (FBS). The culture was incubated at 37°C in 7% CO_2._ The cells began to adhere after 24 to 72 hours and spread to the surface of the flask. Both FAP and control samples were treated with the same culture conditions, including timing for passaging and harvesting. Importantly, all the samples were deidentified, including notation on carrier or control status, and no significant difference in growth or apoptosis among them was noted. At harvest, all the cultures were in log phase. Cells were harvested at passage 1 or 7 by washing once with 5 ml of phosphate buffered saline (PBS) Ca^++^ and Mg^++^, then adding 3 ml of 0.04% trypsin for approximately 5 minutes or until the cells lifted. After adding 5 ml of culture media to stop the trypsin, the cells were transferred to a 15 ml tube, pelleted at 900 rpm, and washed twice with 10 ml of PBS. The cells were counted by using a hemocytometer. The final pellet contained about 10 to 15 million cells.

### 2D gel proteomics

Gels of three overlapping pH ranges (pH 4—7, 5—8, and 6—11) were used to display more than 4,000 protein spots for each sample (Figure 1). The first dimension was performed using IPG strips of pH 4—7, 5—8 (ReadyStrip; 0.5 × 3 × 170 mm, Bio-Rad, Hercules, CA, USA) and 6—11 (Immobiline™ DryStrip; 0.5 × 3 × 180 mm, Amersham Pharmacia Biotech, Uppsala, Sweden) on Protean IEF Cell System (Bio-Rad). The second dimension was 12% polyacrylamide gel (acrylamide/bisacrylamide 37.5:1, 2.6% cross-linker; gel dimension, 20 cm × 20 cm × 1 mm). All gels in this study were run in quadruplicates and the two best gels of each sample were used for image analysis, all according to Standard Operating Procedures [49, 50]. By identifying each protein at least twice from replicated gels, we confirmed the fidelity of our sequence of electrophoresis, image analysis, robotic spot excision, protein digestion, and protein identification by mass spectrometry.

### Immunofluorescence localization of RSU1 protein in colonic fibroblasts

2.5x10^5^ cells were seeded on cover slips in 6 cm tissue culture dish overnight. Cells were then washed 3X with PBS, fixed in ice-cold methanol, and permeabilized with 0.1 % Triton X-100 in PBS. Cells were incubated in 2% milk in PBS for 1 hour and then probed with antibodies anti-APC (ab15270, rabbit polyclonal, 1:20 to 1:100, Abcam, MA) and anti-RSU1 (chicken polyclonal, 1:100-1:1000, GenWay, CA), in antibody dilution buffer (0.2% milk in PBS). After three washes with PBS, the cells were incubated with species-specific secondary antibodies conjugated with Alexa Fluor 488, 546, or 555 (Molecular Probes, CA). The cover slips were mounted on glass slides with mounting medium (0.5% N-Propyl Gallate, 90% glycerol in PBS), sealed with nail polish, and subjected to Z-axis optical sectioning (0.5-0.75 μM/section) in a Nikon E800 upright microscope with Zeiss Radiance 2000 confocal scanhead using LaserSharp 2000 software (Bio-Rad, CA).

### Real-time PCR

Contaminating DNA in RNA preparations was removed using TURBO DNA-*free*™ (Ambion). RNA was quantified using the Agilent 2100 BioAnalyzer in combination with a RNA 6000 Nano LabChip. RNA was reverse-transcribed using M-MLV reverse transcriptase (Ambion) and a mixture of anchored oligo-dT and random decamers. Using Taqman chemistry, 5'-nuclease assays were run on a 7900 HT sequence detection system (Applied Biosystems). Taqman sets were designed using Primer Express™ version 2.0 software from Applied Biosystems. The 5' and 3' probe ends were labeled with reporter dye 6-FAM (6-carboxy-fluorescein, Glenn Research) and quencher dye BHQ1 (Black Hole Quencher, Biosearch Technologies), respectively. All primers and probes were synthesized by the Fox Chase Cancer Center Fannie Rippel Biochemistry and Biotechnology Facility. PCR master mix from Eurogentec was used for PCR. Primer and probe concentrations were 400 nM and 100 nM respectively. Cycling conditions were 95°C, for 15 min, followed by 40 (2-steps) cycles (95°C, 15 sec; 60°C, 60 sec). Cycle threshold (Ct) values were converted to quantities (in arbitrary units) using a standard curve (4 points, 5-fold serial dilutions) established with a calibrator sample. For each sample, 2 RT reactions were performed with inputs of 50 and 10 ng RNA. An aliquot of the cDNA was used for PCR. The assay components were: DJ-1: forward GATACTGAAGGAGCAGGAAAACC, reverse TGAGCCAACAGAGCAGTAGGA, probe (6-FAM)CCTGATAGCCGCCATCTGTGCAG(BHQ1). RSU1: forward GTCATTATTCTTCTTCGGTGGTTCT, reverse TCCGTTCTGAGACATACAAATACCT, probe (6-FAM)TGGCCTGCATGTGTCTGCCGTA(BHQ1).

### Statistical analyses

Statistical analysis of 2D gel images using 16 bit pixel intensity information was supported by Progenesis Discovery Workstation software (Nonlinear Dynamics, Inc.). Results were edited by an experienced operator. With 12 gels in the image analysis of pH 5—8 range, we rejected any spot that was missing in more than 2 gels. The dynamic range of the spot intensities in this presentation was about 600. A cut-off value for 1.5 fold change was used to output a list of significant protein changes.

### Protein identification

Robotic cutting of protein spots, trypsin digestion, automated MALDI-TOF mass spectrometry, and protein identification from Swiss-Prot database were previously described in detail [49, 50]. The identity and function of the genes separating FAP fibroblasts from control were explored at the gene ontology databases FatiGO+(http://babelomics.bioinfo.cipf.es/functional.html) and Database for Annotation, Visualization and Integrated Discovery (DAVID http://david.abcc.ncifcrf.gov/). The fibroblast differential proteome was further compared with the FAP crypt differential proteome [4] to determine protein changes in common between crypt and fibroblast that may suggest protein changes related to *APC* heterozygous mutation.

### Western blotting validation of biomarkers

Western blotting in samples of colonic crypt cells and colonic primary fibroblast cultures were performed for RSU1 and DJ-1 to validate their signatures as primary proteomic alterations in the FAP heterozygous state. For each sample, 100 μg of protein was resolved on a 1D or a 2D gel. Blotting was performed at 200 mA for 2 hours. Antibodies used were Anti-RSU1 (chicken polyclonal, 1:100—1:1000, GenWay, CA) and anti-DJ-1 (E2.1, monoclonal, 1:10, Zymed, Inc., CA). Blocking was done in 5% non-fat dry milk/TBS, first antibody anti-DJ-1 1:200, second antibody goat anti-mouse (Zymed Inc) 1:2000. Imaging was obtained on Kodak BioMax film developed with Bio-Rad ECL reagent.

## SUPPLEMENTAL DATA




























