# Supplementary material for: APC+/− alters colonic fibroblast proteome in FAP
Source: Oncotarget. 2011 Mar 15;2(3):197–208. doi: 10.18632/oncotarget.241 (PMC3195363; doi:10.18632/oncotarget.241)
Supplement: Supplementary file 1 [file oncotarget-02-197-s001.doc]

| **Sample Identification No.** | **Gender** | **Age** | ***APC* Mutation** | **Diagnosis** |
| --- | --- | --- | --- | --- |
| **FAP Patients** |  |  |  |  |
| 317 | F | 23 | Not detected | FAP |
| 344 | M | 42 | Not detected | FAP |
| 484 | M | 53 | Before exon 4 (Nonconsc IVS) (Genetically attenuated) | FAP |
| 514 | F | 17 | Codon 1148 | FAP |
| 516 | F | 39 | Codon 178 (Genetically attenuated) | FAP |
| 548 | F | 45 | Codon 953 | FAP |
| 549 | F | 34 | Codon 216 | FAP |
| 601 | M | 48 | IVS4+1>A | FAP |
| 602 | M | 42 | APC3714delT | FAP |
| 608 | M | 24 | APC3927del5 | FAP |
| 622 | M | 26 | APC3183del5 | FAP |
| **Controls** |  |  |  |  |
| 340 | M | 73 |  | No evidence of disease |
| 461 | F | 53 |  | No evidence of disease |
| 471 | F | 54 |  | No evidence of disease |
| 508 | unknown | unknown |  | unknown |
| 507 | unknown | unknown |  | unknown |
| 509 | unknown | unknown |  | unknown |
| 513 | unknown | unknown |  | unknown |
| 129 | M | 47 |  | Rectal Cancer |
| 70 | M | 54 |  | Rectal Cancer |
| 203 | M | 49 |  | Rectal Cancer |
| 144 | M | 58 |  | Rectal Cancer |
| 162 | F | 56 |  | Rectal Prolapse |
| 73 | F | 56 |  | Sigmoid Cancer |
| 206 | F | 41 |  | Rectal Cancer |
| 233 | F | 47 |  | Rectal Cancer |

**Supplemental Data 1.** Patient samples used in the comparison of the colon crypt proteomes of FAP patients and controls (distal mucosa from sporadic cancer cases).
