# Supplementary material for: APC+/− alters colonic fibroblast proteome in FAP
Source: Oncotarget. 2011 Mar 15;2(3):197–208. doi: 10.18632/oncotarget.241 (PMC3195363; doi:10.18632/oncotarget.241)
Supplement: Supplementary file 3 [file oncotarget-02-197-s003.doc]

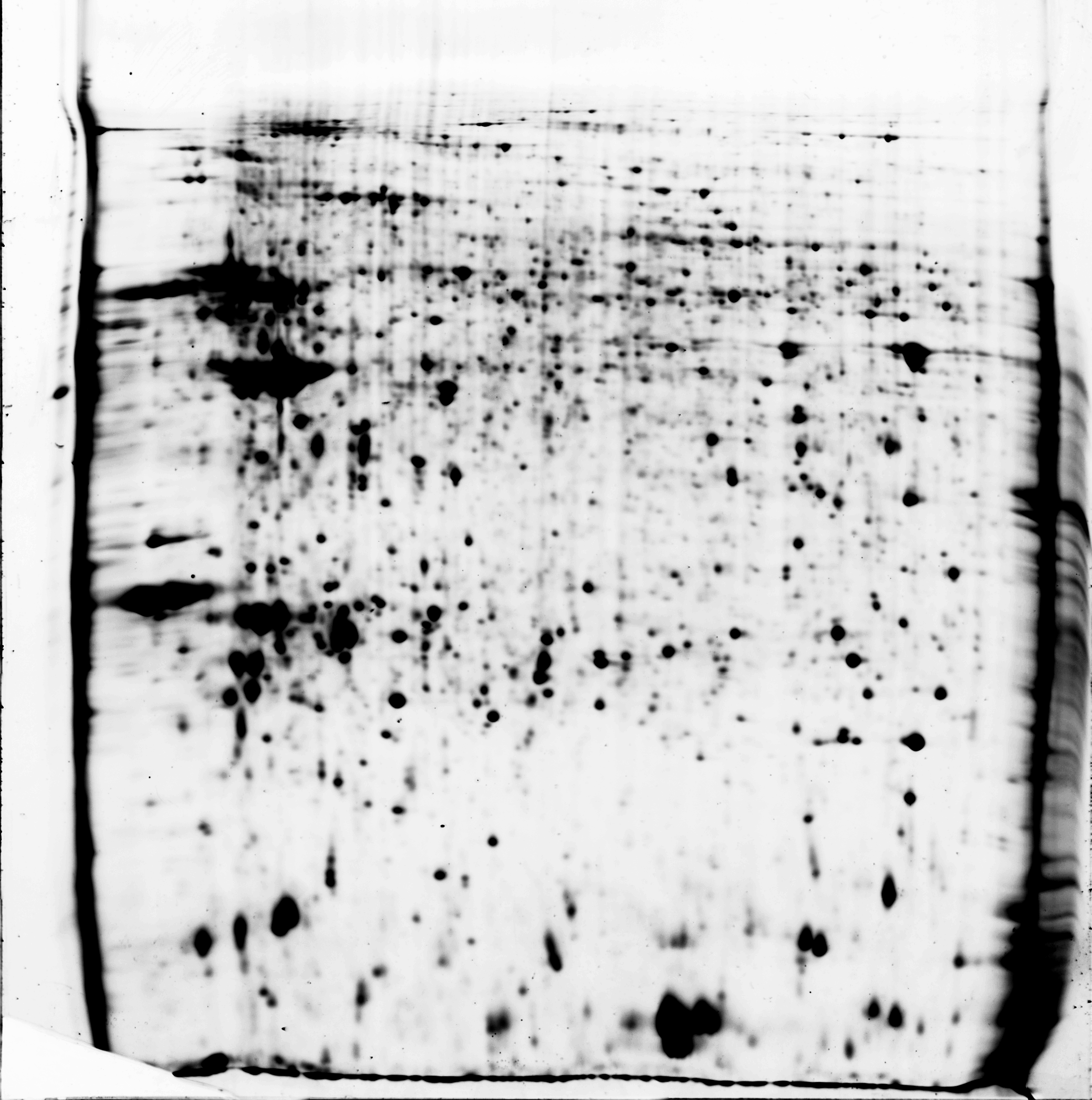


**Molecular Weight**

**120K**

**20K**

**5**

**8**

**pH**

**Supplemental Data 3**. Searchable, Point & Click **pH 5-8 2D gel map of**

**human colonic Fibroblast** with protein identification numbers, protein

names, and hyperlinks to gene ontology. Please note that the point and

click features do not work in pdf, but functional files are provided at

our web site.
