# Supplementary material for: APC+/− alters colonic fibroblast proteome in FAP
Source: Oncotarget. 2011 Mar 15;2(3):197–208. doi: 10.18632/oncotarget.241 (PMC3195363; doi:10.18632/oncotarget.241)
Supplement: Supplementary file 5 [file oncotarget-02-197-s005.doc]

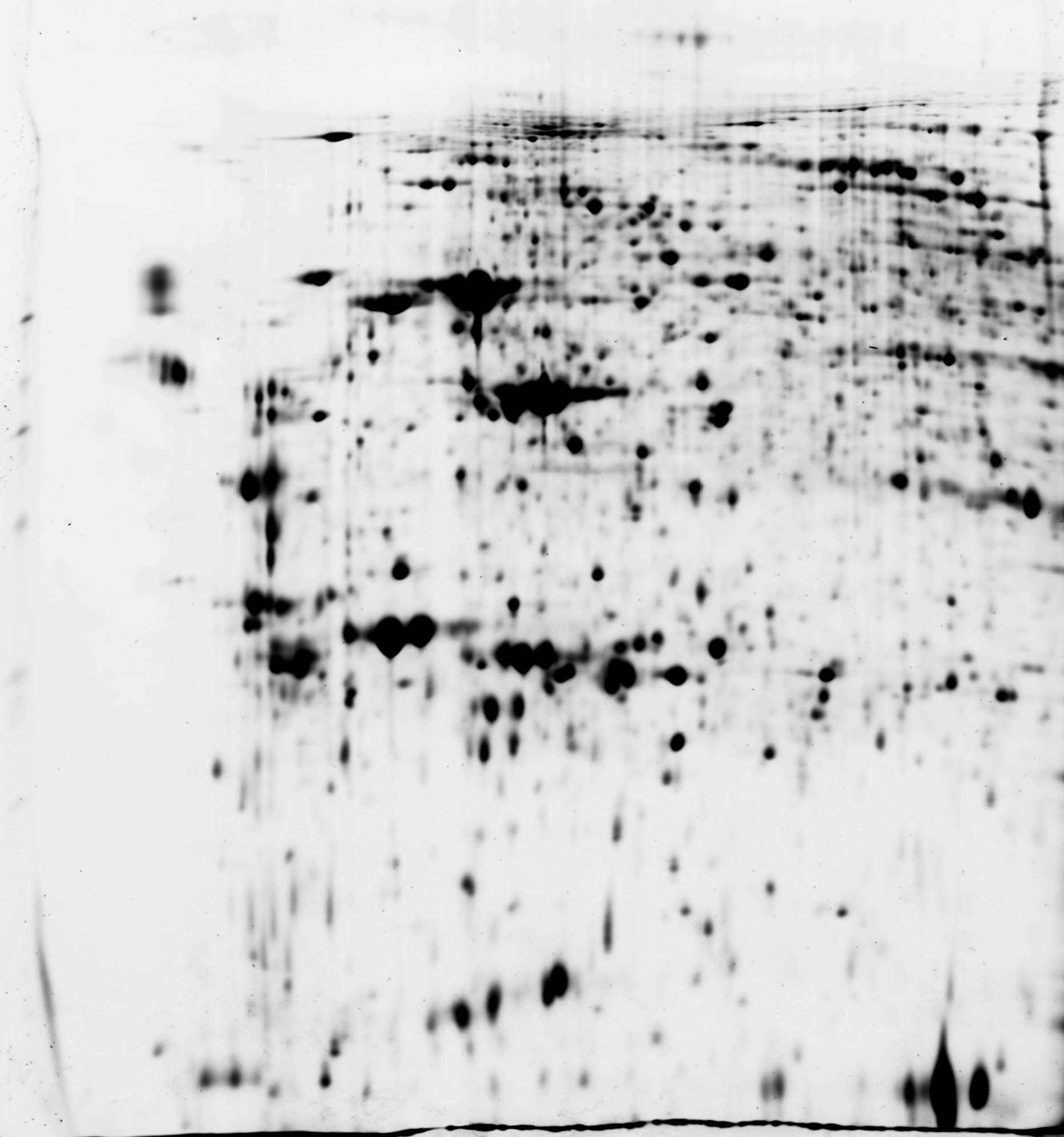


**Molecular Weight**

396

429

561

510

222

1321

1124

1183

870

704

489

353

653

835

1293

961

894

936

1099

532

395

1358

750

1448

1137

889

853

1878

1815

925

1844

1784

1690

1810

1755

1877

1669

1897

1744

1500

1191

980

661

935

1342

1082

2085

927

1081

1459

1199

1797

2232

2549

1763

1305

1618

2264

2397

2592

1761

1403

2270

2318

2574

1430

1819

2388

2410

1736

2430

2803

3169

3156

1470

1559

1333

**120K**

**20K**

**4**

**7**

**pH**

**Supplemental Data 5.** Searchable, Point & Click **pH 4-7 2D gel protein**

**differences map of human colonic Fibroblast** with protein identification

numbers, protein names, and hyperlinks to gene ontology. Please note

that the point and click features do not work in pdf, but functional files are

provided at our web site.
