# Supplementary material for: APC+/− alters colonic fibroblast proteome in FAP
Source: Oncotarget. 2011 Mar 15;2(3):197–208. doi: 10.18632/oncotarget.241 (PMC3195363; doi:10.18632/oncotarget.241)
Supplement: Supplementary file 6 [file oncotarget-02-197-s006.doc]

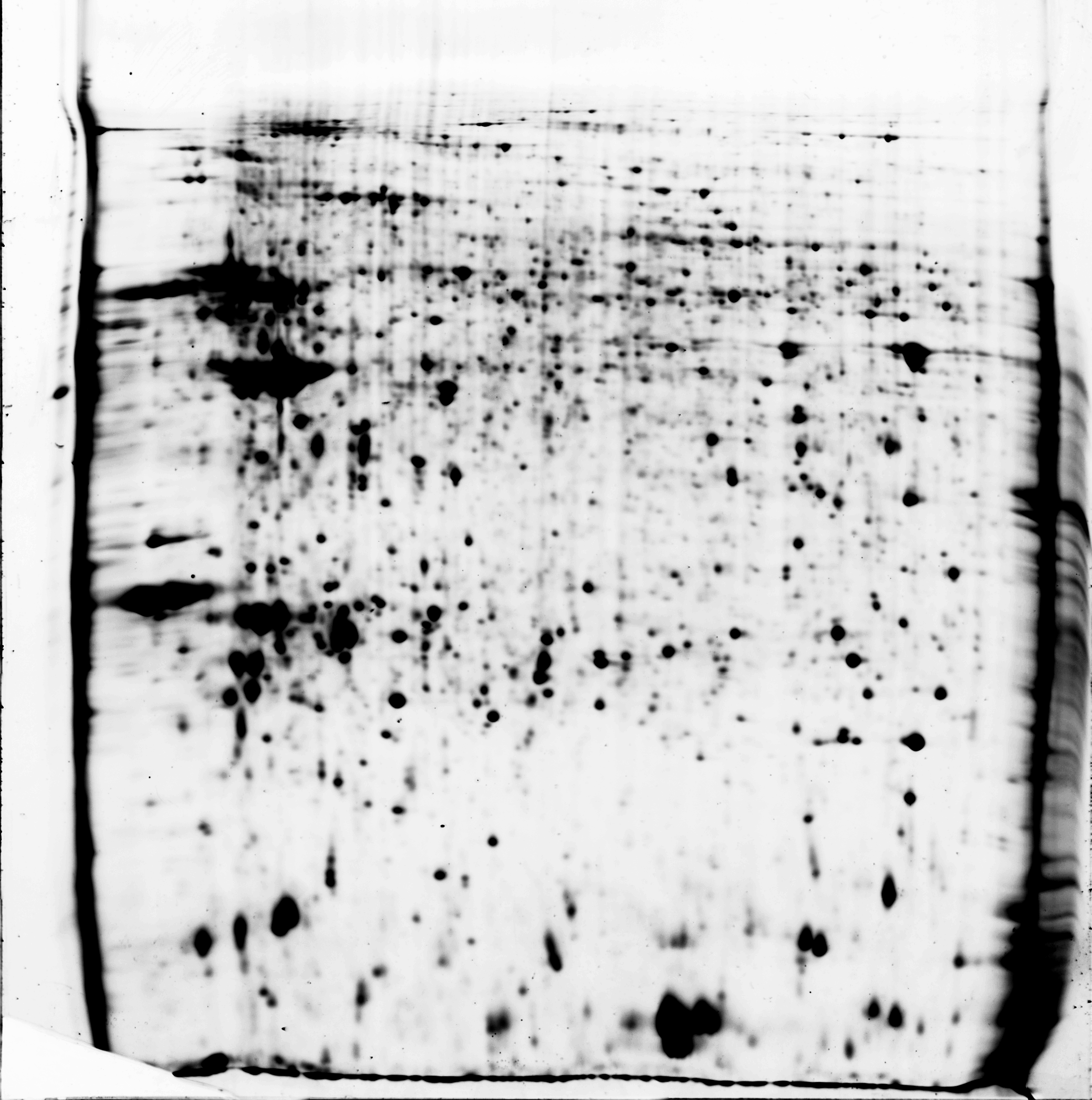


**Molecular Weight**

338

316

260

863

207

525

320

331

350

397

1485

996

781

1286

1336

2042

1303

2011

1315

1153

763

1062

1641

2357

1419

1591

1252

790

2493

2537

2595

2484

2606

2446

3170

3219

**120K**

**20K**

**5**

**8**

**pH**

**Supplemental Data 6.** Searchable, Point & Click **pH 5-8 2D gel protein**

**differences map of human colonic Fibroblast** with protein identification

numbers, protein names, and hyperlinks to gene ontology. Please note that

the point and click features do not work in pdf, but functional files are

provided at our web site.
