# Supplementary material for: APC+/− alters colonic fibroblast proteome in FAP
Source: Oncotarget. 2011 Mar 15;2(3):197–208. doi: 10.18632/oncotarget.241 (PMC3195363; doi:10.18632/oncotarget.241)
Supplement: Supplementary file 7 [file oncotarget-02-197-s007.doc]

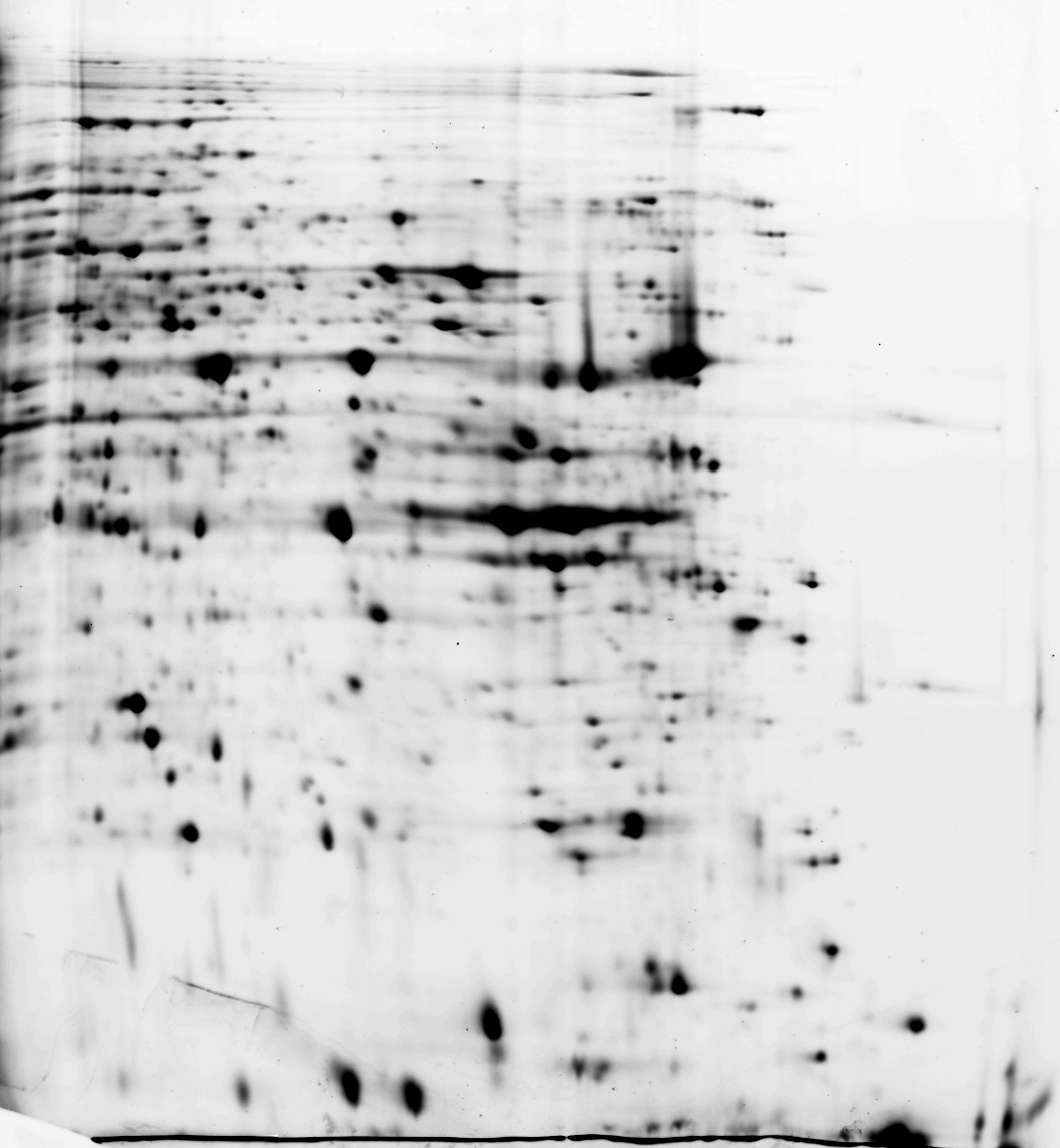


**Molecular Weight**

838

780

527

723

719

901

1118

1198

880

870

906

1048

1049

658

1256

1310

1311

1419

1478

1538

1578

1755

1637

1752

1831

2025

2077

2096

2205

2089

2207

2147

2165

2306

2307

**120K**

**20K**

**6**

**11**

**pH**

**Supplemental Data 7.** Searchable, Point & Click **pH 6-11 2D gel protein**

**differences map of human colonic Fibroblast** with protein identification

numbers, protein names, and hyperlinks to gene ontology. Please note that

the point and click features do not work in pdf, but functional files are

provided at our web site.
