# Supplementary material for: APC+/− alters colonic fibroblast proteome in FAP
Source: Oncotarget. 2011 Mar 15;2(3):197–208. doi: 10.18632/oncotarget.241 (PMC3195363; doi:10.18632/oncotarget.241)
Supplement: Supplementary file 8 [file oncotarget-02-197-s008.doc]

| **FAP >2X Control** | | | | | | | | | |
| --- | --- | --- | --- | --- | --- | --- | --- | --- | --- |
| **FAP fold change from Progenesis, control=1** | **pH range specific Unique ID** | **2D gel pH range** | **Swiss-Prot ID #** | **Protein Name** | **Theoretical pI** | **Theoretical MW** | **MASCOT Score** | **Amino Acid Coverage** | **Swiss-Prot Accession #** |
| 22.6 | 395 | 4-7 | P13639 | Elongation factor 2 (EF-2) | 6.4 | 95207 | 163 | 35 | EF2_HUMAN |
| 10.5 | 1358 | 4-7 | P06733 | Alpha enolase | 7.0 | 47038 | 168 | 49 | ENOA_HUMAN |
| 9.1 | 1755 | 4-7 | Q14847 | LIM and SH3 domain protein 1 (LASP-1) | 6.6 | 29717 | 95 | 44 | LASP1_HUMAN |
| 6.2 | 429 | 4-7 | O43707 | Alpha-actinin 4 (F-actin cross linking protein) | 5.3 | 104854 | 82 | 18 | ACTN4_HUMAN |
| 5.8 | 1191 | 4-7 | P08729 | Keratin, type II cytoskeletal 7 (CK 7) | 5.5 | 51287 | 211 | 45 | K2C7_HUMAN |
| 4.2 | 1797 | 4-7 | Q05682 | Caldesmon (CDM) | 5.6 | 93250 | 63 | 13 | CALD1_HUMAN |
| 4.2 | 1797 | 4-7 | P07237 | Protein disulfide-isomerase precursor | 4.7 | 55294 | 192 | 36 | PDIA1_HUMAN |
| 4.1 | 2089 | 5-8 | P02511 | Alpha crystallin B | 6.8 | 20159 | 138 | 46 | CRYAB_HUMAN |
| 4.0 | 532 | 4-7 | Q05682 | Caldesmon (CDM) | 5.6 | 93250 | 101 | 29 | CALD1_HUMAN |
| 3.6 | 2307 | 5-8 | P62937 | Peptidyl-prolyl cis-trans isomerase A | 7.8 | 17881 | 57 | 35 | PPIA_HUMAN |
| 3.4 | 1403 | 4-7 | Q96D15 | Reticulocalbin-3 | 4.7 | 35206 | 48 | 26 | RCN3_HUMAN |
| 3.4 | 1403 | 4-7 | P08670 | Vimentin | 5.1 | 53520 | 75 | 20 | VIME_HUMAN |
| 3.4 | 1342 | 5-8 | P04406 | Glyceraldehyde-3-phosphate dehydrogenase, | 8.6 | 35922 | 108 | 55 | G3P_HUMAN |
| 3.4 | 1430 | 4-7 | P08670 | Vimentin | 5.1 | 53520 | 219 | 55 | VIME_HUMAN |
| 3.4 | 935 | 4-7 | Q02818 | Nucleobindin 1 (CALNUC) | 5.1 | 51146 | 115 | 33 | NUCB1_HUMAN |
| 3.3 | 489 | 4-7 | Q05682 | Caldesmon (CDM) | 5.6 | 93250 | 194 | 37 | CALD1_HUMAN |
| 3.2 | 2803 | 4-7 | P07237 | Protein disulfide-isomerase | 4.7 | 55294 | 49 | 14 | PDIA1_HUMAN |
| 3.1 | 1761 | 4-7 | P07437 | Tubulin beta-2 chain | 4.8 | 49671 | 93 | 19 | TBB2_HUMAN |
| 3.1 | 880 | 5-8 | P34897 | Serine hydroxymethyltransferase, mitochondrial | 8.1 | 52560 | 108 | 23 | GLYM_HUMAN |
| 3.0 | 1763 | 4-7 | Q9BQE3 | Tubulin alpha-6 chain | 5.0 | 49895 | 84 | 27 | TBA6_HUMAN |
| 2.9 | 1637 | 5-8 | P04083 | Annexin A1 | 6.6 | 38583 | 94 | 35 | ANXA1_HUMAN |
| 2.9 | 2081 | 4-7 | P10768 | Esterase D | 6.5 | 31463 | 62 | 28 | ESTD_HUMAN |
| 2.9 | 1844 | 4-7 | Q14847 | LIM and SH3 domain protein 1 (LASP-1) | 6.6 | 29717 | 91 | 52 | LASP1_HUMAN |
| 2.9 | 353 | 4-7 | O00469 | Procollagen-lysine,2-oxoglutarate 5-dioxygenase 2 | 6.2 | 82029 | 118 | 28 | PLOD2_HUMAN |
| 2.9 | 1470 | 4-7 | P08670 | Vimentin | 5.1 | 53520 | 251 | 47 | VIME_HUMAN |
| 2.8 | 2549 | 4-7 | Q99497 | DJ-1 protein | 6.3 | 19891 | 102 | 65 | PARK7_HUMAN |
| 2.7 | 2207 | 5-8 | P50458 | LIM/homeobox protein Lhx2 | 8.8 | 44373 | 56 | 11 | LHX2_HUMAN |
| 2.7 | 2207 | 5-8 | Q01995 | Transgelin | 8.9 | 22480 | 92 | 40 | TAGL_HUMAN |
| 2.6 | 1877 | 4-7 | P06733 | Alpha enolase | 7.0 | 47038 | 75 | 34 | ENOA_HUMAN |
| 2.6 | 661 | 4-7 | P08238 | Heat shock protein HSP 90-beta | 5.0 | 83133 | 100 | 25 | HS90B_HUMAN |
| 2.6 | 661 | 4-7 | P20700 | Lamin B1 | 5.1 | 66277 | 129 | 30 | LMNB1_HUMAN |
| 2.6 | 2388 | 4-7 | P30041 | Peroxiredoxin 6 | 6.0 | 24904 | 100 | 42 | PRDX6_HUMAN |
| 2.5 | 1118 | 5-8 | P00558 | Phosphoglycerate kinase 1 | 8.3 | 44483 | 57 | 24 | PGK1_HUMAN |
| 2.5 | 1559 | 4-7 | P08670 | Vimentin | 5.1 | 53520 | 186 | 30 | VIME_HUMAN |
| 2.5 | 1878 | 4-7 | P04083 | Annexin A1 | 6.6 | 38583 | 178 | 49 | ANXA1_HUMAN |
| 2.4 | 1321 | 4-7 | P26641 | Elongation factor 1-gamma (EF-1-gamma) | 6.3 | 49988 | 153 | 25 | EF1G_HUMAN |
| 2.4 | 1321 | 4-7 | Q13418 | Integrin-linked protein kinase | 8.3 | 51419 | 75 | 23 | ILK1_HUMAN |
| 2.4 | 302 | 4-7 | P41250 | Glycyl-tRNA synthetase | 6.6 | 83140 | 102 | 19 | SYG_HUMAN |
| 2.4 | 1500 | 4-7 | P11021 | 78 kDa glucose-regulated protein | 5.0 | 70479 | 62 | 19 | GRP78_HUMAN |
| 2.3 | 1810 | 4-7 | Q13347 | Eukaryotic translation initiation factor 3 subunit 2 (eIF-3 beta) | 5.4 | 36502 | 84 | 40 | IF32_HUMAN |
| 2.3 | 1784 | 4-7 | Q9UJ70 | N-acetylglucosamine kinase | 5.8 | 37244 | 59 | 22 | NAGK_HUMAN |
| 2.3 | 653 | 4-7 | P26038 | Moesin | 6.1 | 67689 | 200 | 45 | MOES_HUMAN |
| 2.3 | 1819 | 4-7 | P09493 | Tropomyosin 1 alpha chain | 4.7 | 32709 | 198 | 54 | TPM1_HUMAN |
| 2.3 | 2264 | 4-7 | O95865 | NG,NG-dimethylarginine dimethylaminohydrolase 2 | 5.7 | 29644 | 64 | 31 | DDAH2_HUMAN |
| 2.2 | 1690 | 4-7 | P07355 | Annexin A2 | 7.6 | 38473 | 232 | 53 | ANXA2_HUMAN |
| 2.2 | 1690 | 4-7 | Q15019 | Septin-2 (NEDD5 protein homolog) | 6.2 | 41487 | 75 | 31 | SEPT2_HUMAN |
| 2.1 | 2592 | 4-7 | Q01105 | SET protein (Phosphatase 2A inhibitor I2PP2A) | 4.2 | 33489 | 54 | 21 | SET_HUMAN |
| 2.1 | 510 | 4-7 | Q15942 | Zyxin 2 | 6.2 | 61277 | 60 | 23 | ZYX_HUMAN |
| 2.1 | 980 | 4-7 | P11021 | 78 kDa glucose-regulated protein | 5.0 | 70479 | 76 | 18 | GRP78_HUMAN |
| 2.1 | 870 | 4-7 | P02545 | Lamin A/C | 6.6 | 74139 | 211 | 43 | LMNA_HUMAN |
| 2.0 | 1333 | 4-7 | P17980 | 26S protease regulatory subunit 6A (TAT-binding protein 1) (TBP-1) | 5.1 | 49204 | 67 | 24 | PRS6A_HUMAN |
| 2.0 | 1448 | 4-7 | P61163 | Alpha-centractin (Centractin) (ARP1) | 6.2 | 42614 | 88 | 34 | ACTZ_HUMAN |
| 2.0 | 936 | 4-7 | P26038 | Moesin | 6.1 | 67689 | 72 | 25 | MOES_HUMAN |
| **Proteins Found Only in FAP** | | | | | | | | | |
| - | 2232 | 4-7 | P62736 | Actin, aortic smooth muscle (Alpha-actin-2) | 5.2 | 41775 | 57 | 29 | ACTA_HUMAN |
| - | 1669 | 4-7 | P60709 | Actin, cytoplasmic 1 (Beta-actin) | 5.3 | 41606 | 60 | 22 | ACTB_HUMAN |
| - | 2232 | 4-7 | P60709 | Actin, cytoplasmic 1 (Beta-actin) | 5.3 | 41606 | 78 | 37 | ACTB_HUMAN |
| - | 750 | 4-7 | Q05682 | Caldesmon (CDM) | 5.6 | 93250 | 86 | 25 | CALD1_HUMAN |
| - | 2270 | 4-7 | P07339 | Cathepsin D | 5.6 | 37852 | 49 | 14 | CATD_HUMAN |
| - | 1099 | 4-7 | O43175 | D-3-phosphoglycerate dehydrogenase | 6.3 | 56519 | 69 | 28 | SERA_HUMAN |
| - | 894 | 4-7 | P21333 | Filamin A | 5.7 | 280630 | 67 | 10 | FLNA_HUMAN |
| - | 889 | 4-7 | P02545 | Lamin A/C) | 6.6 | 74139 | 222 | 42 | LMNA_HUMAN |
| - | 1815 | 4-7 | Q14847 | LIM and SH3 domain protein 1 (LASP-1) | 6.6 | 29717 | 66 | 37 | LASP1_HUMAN |
| - | 1124 | 4-7 | P30101 | Protein disulfide-isomerase A3 | 5.6 | 54265 | 94 | 26 | PDIA3_HUMAN |
| - | 925 | 4-7 | P13667 | Protein disulfide-isomerase A4 | 4.9 | 70672 | 79 | 20 | PDIA4_HUMAN |
| - | 1293 | 4-7 | P50395 | Rab GDP dissociation inhibitor beta | 6.1 | 50663 | 124 | 34 | GDIB_HUMAN |
| - | 2430 | 4-7 | P52565 | Rho GDP-dissociation inhibitor 1 | 5.0 | 23076 | 78 | 38 | GDIR_HUMAN |
| - | 835 | 4-7 | P31948 | Stress-induced-phosphoprotein 1 (STI1) (Hsc70/Hsp90-organizing protein) | 6.4 | 62639 | 91 | 24 | STIP1_HUMAN |
| - | 1124 | 4-7 | P78371 | T-complex protein 1, beta subunit (TCP-1-beta) | 6.0 | 57357 | 66 | 18 | TCPB_HUMAN |
| - | 2397 | 4-7 | P60174 | Triosephosphate isomerase | 6.5 | 26538 | 70 | 38 | TPIS_HUMAN |
| - | 1736 | 4-7 | P09493 | Tropomyosin 1 alpha chain (Alpha-tropomyosin) | 4.7 | 32709 | 113 | 38 | TPM1_HUMAN |
| - | 1744 | 4-7 | Q9BQE3 | Tubulin alpha-6 chain (Alpha-tubulin 6) | 5.0 | 49895 | 55 | 20 | TBA6_HUMAN |
| - | 1082 | 4-7 | P07437 | Tubulin beta-2 chain | 4.8 | 49671 | 68 | 21 | TBB2_HUMAN |
| - | 1342 | 4-7 | P07437 | Tubulin beta-2 chain | 4.8 | 49671 | 69 | 21 | TBB2_HUMAN |
| - | 1199 | 4-7 | P08670 | Vimentin | 5.1 | 53520 | 186 | 39 | VIME_HUMAN |
| - | 1459 | 4-7 | P08670 | Vimentin | 5.1 | 53520 | 272 | 58 | VIME_HUMAN |
| - | 2318 | 4-7 | P08670 | Vimentin | 5.1 | 53520 | 56 | 24 | VIME_HUMAN |
| - | 3156 | 4-7 | P08670 | Vimentin | 5.1 | 53520 | 58 | 19 | VIME_HUMAN |
| - | 3169 | 4-7 | P08670 | Vimentin | 5.1 | 53520 | 73 | 21 | VIME_HUMAN |
| - | 222 | 4-7 | P18206 | Vinculin (Metavinculin) | 5.5 | 123668 | 125 | 22 | VINC_HUMAN |
| **Control > 2X FAP** | | | | | | | | | |
| -15.1 | 2574 | 4-7 | P49720 | Proteasome subunit beta type 3 | 6.1 | 22949 | 62 | 35 | PSB3_HUMAN |
| -10.6 | 1752 | 5-8 | P62701 | 40S ribosomal protein S4, X isoform (Single copy abundant mRNA protein) (SCR10) | 10.2 | 29467 | 77 | 37 | RS4X_HUMAN |
| -7.9 | 927 | 4-7 | P61978 | Heterogeneous nuclear ribonucleoprotein K (hnRNP K) | 5.4 | 50976 | 78 | 23 | HNRPK_HUMAN |
| -7.0 | 723 | 5-8 | P14618 | Pyruvate kinase, isozymes M1/M2 | 8.0 | 57806 | 142 | 32 | KPYM_HUMAN |
| -6.3 | 901 | 5-8 | Q9NVA2 | Septin 11 | 6.4 | 49267 | 73 | 23 | SEP11_HUMAN |
| -5.4 | 2205 | 5-8 | P50458 | LIM/homeobox protein Lhx2 | 8.8 | 44373 | 58 | 11 | LHX2_HUMAN |
| -5.4 | 2205 | 5-8 | Q01995 | Transgelin (SM22-alpha) | 8.9 | 22480 | 95 | 42 | TAGL_HUMAN |
| -5.2 | 1310 | 5-8 | Q05682 | Caldesmon (CDM) | 5.6 | 93250 | 53 | 14 | CALD1_HUMAN |
| -4.9 | 1311 | 5-8 | Q05682 | Caldesmon (CDM) | 5.6 | 93250 | 127 | 26 | CALD1_HUMAN |
| -3.8 | 527 | 5-8 | P11940 | Polyadenylate-binding protein 1 (PABP 1) | 9.5 | 70671 | 82 | 19 | PABP1_HUMAN |
| -3.8 | 396 | 4-7 | P12814 | Alpha-actinin 1 | 5.3 | 103058 | 109 | 21 | ACTN1_HUMAN |
| -3.6 | 2110 | 5-8 | Q8IV36 | Protein C17orf28 (Down-regulated in multiple cancers-1) | 5.7 | 88745 | 46 | 8 | CQ028_HUMAN |
| -3.4 | 2085 | 4-7 | P08758 | Annexin A5 (Annexin V) | 4.9 | 35806 | 139 | 52 | ANXA5_HUMAN |
| -3.3 | 1049 | 5-8 | P60709 | Actin, cytoplasmic 1 (Beta-actin) | 5.3 | 41606 | 89 | 32 | ACTB_HUMAN |
| -3.3 | 704 | 4-7 | P38646 | Stress-70 protein, mitochondrial (GRP 75) | 5.4 | 68759 | 199 | 36 | GRP75_HUMAN |
| -3.2 | 658 | 5-8 | P02545 | Lamin A/C (70 kDa lamin) | 6.6 | 74139 | 148 | 31 | LMNA_HUMAN |
| -3.0 | 1538 | 5-8 | P09651 | Heterogeneous nuclear ribonucleoprotein A1 | 9.3 | 38715 | 108 | 33 | ROA1_HUMAN |
| -3.0 | 1618 | 4-7 | P07237 | Protein disulfide-isomerase | 4.7 | 55294 | 64 | 16 | PDIA1_HUMAN |
| -2.9 | 1183 | 4-7 | P55795 | Heterogeneous nuclear ribonucleoprotein H' (hnRNP H') | 5.9 | 49264 | 83 | 25 | HNRH2_HUMAN |
| -2.7 | 853 | 4-7 | P02545 | Lamin A/C (70 kDa lamin) | 6.6 | 74139 | 105 | 29 | LMNA_HUMAN |
| -2.7 | 2077 | 5-8 | Q01995 | Transgelin (SM22-alpha) | 8.9 | 22480 | 56 | 33 | TAGL_HUMAN |
| -2.7 | 2096 | 5-8 | P37802 | Transgelin-2 (SM22-alpha homolog) | 8.5 | 22260 | 68 | 33 | TAGL2_HUMAN |
| -2.7 | 2147 | 5-8 | Q01995 | Transgelin (SM22-alpha) | 8.9 | 22480 | 93 | 40 | TAGL_HUMAN |
| -2.7 | 2165 | 5-8 | Q01995 | Transgelin (SM22-alpha) | 8.9 | 22480 | 115 | 46 | TAGL_HUMAN |
| -2.6 | 780 | 5-8 | P11413 | Glucose-6-phosphate 1-dehydrogenase (G6PD) | 6.4 | 59135 | 85 | 23 | G6PD_HUMAN |
| -2.6 | 1256 | 5-8 | P51991 | Heterogeneous nuclear ribonucleoprotein A3 (hnRNP A3) | 9.1 | 39595 | 62 | 21 | ROA3_HUMAN |
| -2.6 | 561 | 4-7 | P15311 | Ezrin (p81) (Cytovillin) (Villin-2) | 6.0 | 69268 | 150 | 36 | EZRI_HUMAN |
| -2.6 | 1081 | 4-7 | P08670 | Vimentin | 5.1 | 53520 | 148 | 35 | VIME_HUMAN |
| -2.5 | 870 | 5-8 | P25705 | ATP synthase alpha chain, mitochondrial | 8.3 | 55209 | 97 | 23 | ATPA_HUMAN |
| -2.5 | 1578 | 5-8 | O15144 | Actin-related protein 2/3 complex subunit 2 (ARP2/3 complex 34 kDa subunit) | 6.8 | 34333 | 77 | 35 | ARPC2_HUMAN |
| -2.5 | 2230 | 5-8 | P50458 | LIM/homeobox protein Lhx2 | 8.8 | 44373 | 55 | 11 | LHX2_HUMAN |
| -2.4 | 1048 | 5-8 | P63267 | Actin, gamma-enteric smooth muscle (Alpha-actin 3) | 5.3 | 41643 | 81 | 24 | ACTH_HUMAN |
| -2.4 | 2410 | 4-7 | Q13162 | Peroxiredoxin 4 | 5.9 | 30540 | 60 | 30 | PRDX4_HUMAN |
| -2.3 | 838 | 5-8 | O15205 | Ubiquitin-like protein FAT10 (Diubiquitin) | 9.2 | 18457 | 53 | 42 | UBD_HUMAN |
| -2.2 | 719 | 5-8 | P14618 | Pyruvate kinase, isozymes M1/M2 | 8.0 | 57806 | 175 | 36 | KPYM_HUMAN |
| -2.2 | 1755 | 5-8 | P62701 | 40S ribosomal protein S4, X isoform (SCR10) | 10.2 | 29467 | 51 | 21 | RS4X_HUMAN |
| -2.2 | 1137 | 4-7 | P14868 | Aspartyl-tRNA synthetase (AspRS) | 6.1 | 57136 | 84 | 26 | SYD_HUMAN |
| -2.1 | 1305 | 4-7 | P08670 | Vimentin | 5.1 | 53520 | 93 | 28 | VIME_HUMAN |
| -2.1 | 1831 | 5-8 | P60174 | Triosephosphate isomerase | 6.5 | 26538 | 81 | 40 | TPIS_HUMAN |
| -2.1 | 961 | 4-7 | P26038 | Moesin | 6.1 | 67689 | 62 | 20 | MOES_HUMAN |
| -2.0 | 1897 | 4-7 | P52907 | F-actin capping protein alpha-1 subunit (CapZ alpha-1) | 5.5 | 32792 | 62 | 37 | CAZA1_HUMAN |
| -2.0 | 1419 | 5-8 | P07355 | Annexin A2 (Annexin II) | 7.6 | 38473 | 104 | 34 | ANXA2_HUMAN |
| -2.0 | 2025 | 5-8 | Q01995 | Transgelin | 8.9 | 22480 | 103 | 51 | TAGL_HUMAN |
| **Proteins Found Only in Control** | | | | | | | | | |
| - | 1188 | 5-8 | P04075 | Fructose-bisphosphate aldolase A | 8.4 | 39289 | 54 | 18 | ALDOA_HUMAN |
| - | 1198 | 5-8 | P04075 | Fructose-bisphosphate aldolase A | 8.4 | 39289 | 55 | 18 | ALDOA_HUMAN |
| - | 1173 | 5-8 | O00410 | Importin beta-3 (RanBP5) | 4.8 | 123499 | 55 | 11 | IMB3_HUMAN |
| - | 906 | 5-8 | P57043 | Integrin-linked protein kinase 2 (ILK-2) | 8.3 | 51393 | 70 | 21 | ILK2_HUMAN |
| - | 1478 | 5-8 | P00338 | L-lactate dehydrogenase A chain (LDH-A) | 8.5 | 36558 | 117 | 35 | LDHA_HUMAN |
| - | 1822 | 5-8 | P18669 | Phosphoglycerate mutase 1 | 6.8 | 28673 | 49 | 24 | PGAM1_HUMAN |
| - | 1173 | 5-8 | Q9Y617 | Phosphoserine aminotransferase (PSAT) | 7.6 | 40423 | 114 | 32 | SERC_HUMAN |
| - | 2306 | 5-8 | P98179 | Putative RNA-binding protein 3 (RNPL) | 8.9 | 17170 | 59 | 47 | RBM3_HUMAN |

**Supplemental Data 8.** Protein expression differences between FAP and normal colonic fibroblast primary cultures.
