# Supplementary material for: APC+/− alters colonic fibroblast proteome in FAP
Source: Oncotarget. 2011 Mar 15;2(3):197–208. doi: 10.18632/oncotarget.241 (PMC3195363; doi:10.18632/oncotarget.241)
Supplement: Supplementary file 9 [file oncotarget-02-197-s009.doc]

| **FAP fold change from Progenesis, control=1** | **pH range specific Unique No.** | **2D gel pH range** | **Swiss-Prot Accession No.** | **Protein Name** | **Theoretical pI** | **Theoretical MW** | **MASCOT Score** | **Amino Acid Coverage (%)** |
| --- | --- | --- | --- | --- | --- | --- | --- | --- |
| FAP Only | 2232 | 4-7 | P62736 | Actin, aortic smooth muscle | 5.2 | 41775 | 57 | 29 |
| -2.4 | 1048 | 5-8 | P63267 | Actin, gamma-enteric smooth muscle | 5.3 | 41643 | 81 | 24 |
| 2 | 1448 | 4-7 | P61163 | Alpha-centractin (Centractin) | 6.2 | 42614 | 88 | 34 |
| 2.9 | 1637 | 5-8 | P04083 | Annexin A1 (Annexin I) | 6.6 | 38583 | 94 | 35 |
| 2.5 | 1878 | 4-7 | P04083 | Annexin A1 (Annexin I) | 6.6 | 38583 | 178 | 49 |
| 4.2 | 1797 | 4-7 | Q05682 | Caldesmon (CDM) | 5.6 | 93250 | 63 | 13 |
| 4 | 532 | 4-7 | Q05682 | Caldesmon (CDM) | 5.6 | 93250 | 101 | 29 |
| 3.3 | 489 | 4-7 | Q05682 | Caldesmon (CDM) | 5.6 | 93250 | 194 | 37 |
| FAP Only | 750 | 4-7 | Q05682 | Caldesmon (CDM) | 5.6 | 93250 | 86 | 25 |
| -5.2 | 1310 | 5-8 | Q05682 | Caldesmon (CDM) | 5.6 | 93250 | 53 | 14 |
| -4.9 | 1311 | 5-8 | Q05682 | Caldesmon (CDM) | 5.6 | 93250 | 127 | 26 |
| 2.1 | 870 | 4-7 | P02545 | Lamin A/C (70 kDa lamin) | 6.6 | 74139 | 211 | 43 |
| FAP Only | 889 | 4-7 | P02545 | Lamin A/C (70 kDa lamin) | 6.6 | 74139 | 222 | 42 |
| -3.2 | 658 | 5-8 | P02545 | Lamin A/C (70 kDa lamin) | 6.6 | 74139 | 148 | 31 |
| -2.7 | 853 | 4-7 | P02545 | Lamin A/C (70 kDa lamin) | 6.6 | 74139 | 105 | 29 |
| 2.6 | 661 | 4-7 | P20700 | Lamin B1 | 5.1 | 66277 | 129 | 30 |
| -6.3 | 901 | 5-8 | Q9NVA2 | Septin 11 | 6.4 | 49267 | 73 | 23 |
| 2.2 | 1690 | 4-7 | Q15019 | Septin-2 (NEDD5 protein homolog) | 6.2 | 41487 | 75 | 31 |
| 2.3 | 1819 | 4-7 | P09493 | Tropomyosin 1 alpha chain | 4.7 | 32709 | 198 | 54 |
| FAP Only | 1736 | 4-7 | P09493 | Tropomyosin 1 alpha chain | 4.7 | 32709 | 113 | 38 |
| FAP Only | 1082 | 4-7 | P07437 | Tubulin beta-2 chain | 4.8 | 49671 | 68 | 21 |
| FAP Only | 1342 | 4-7 | P07437 | Tubulin beta-2 chain | 4.8 | 49671 | 69 | 21 |
| 3.1 | 1761 | 4-7 | P07437 | Tubulin beta-2 chain | 4.8 | 49671 | 93 | 19 |
| **Proteins involved in Cytoskeleton organization and biogenesis** | | | | | | | | |
| FAP Only | 1669 | 4-7 | P60709 | Actin, cytoplasmic 1 (Beta-actin) | 5.3 | 41606 | 60 | 22 |
| FAP Only | 2232 | 4-7 | P60709 | Actin, cytoplasmic 1 (Beta-actin) | 5.3 | 41606 | 78 | 37 |
| -3.3 | 1049 | 5-8 | P60709 | Actin, cytoplasmic 1 (Beta-actin) | 5.3 | 41606 | 89 | 32 |
| -2.5 | 1578 | 5-8 | O15144 | Actin-related protein 2/3 complex subunit 2 | 6.8 | 34333 | 77 | 35 |
| -2 | 1897 | 4-7 | P52907 | F-actin capping protein alpha-1 subunit | 5.5 | 32792 | 62 | 37 |
| 5.8 | 1191 | 4-7 | P08729 | Keratin, type II cytoskeletal 7 | 5.5 | 51287 | 211 | 45 |
| 9.1 | 1755 | 4-7 | Q14847 | LIM and SH3 domain protein 1 (LASP-1) | 6.6 | 29717 | 95 | 44 |
| 2.9 | 1844 | 4-7 | Q14847 | LIM and SH3 domain protein 1 (LASP-1) | 6.6 | 29717 | 91 | 52 |
| FAP Only | 1815 | 4-7 | Q14847 | LIM and SH3 domain protein 1 (LASP-1) | 6.6 | 29717 | 66 | 37 |
| 3 | 1763 | 4-7 | Q9BQE3 | Tubulin alpha-6 chain (Alpha-tubulin 6) | 5 | 49895 | 84 | 27 |
| FAP Only | 1744 | 4-7 | Q9BQE3 | Tubulin alpha-6 chain (Alpha-tubulin 6) | 5 | 49895 | 55 | 20 |
| 3.4 | 1403 | 4-7 | P08670 | Vimentin | 5.1 | 53520 | 75 | 20 |
| 3.4 | 1430 | 4-7 | P08670 | Vimentin | 5.1 | 53520 | 219 | 55 |
| 2.9 | 1470 | 4-7 | P08670 | Vimentin | 5.1 | 53520 | 251 | 47 |
| 2.5 | 1559 | 4-7 | P08670 | Vimentin | 5.1 | 53520 | 186 | 30 |
| -2.1 | 1305 | 4-7 | P08670 | Vimentin | 5.1 | 53520 | 93 | 28 |
| -2.6 | 1081 | 4-7 | P08670 | Vimentin | 5.1 | 53520 | 148 | 35 |
| FAP Only | 1199 | 4-7 | P08670 | Vimentin | 5.1 | 53520 | 186 | 39 |
| FAP Only | 1459 | 4-7 | P08670 | Vimentin | 5.1 | 53520 | 272 | 58 |
| FAP Only | 2318 | 4-7 | P08670 | Vimentin | 5.1 | 53520 | 56 | 24 |
| FAP Only | 3156 | 4-7 | P08670 | Vimentin | 5.1 | 53520 | 58 | 19 |
| FAP Only | 3169 | 4-7 | P08670 | Vimentin | 5.1 | 53520 | 73 | 21 |
| **Proteins link Cytoskeleton to Plasma Membrane** | | | | | | | | |
| -3.8 | 396 | 4-7 | P12814 | Alpha-actinin 1 | 5.3 | 103058 | 109 | 21 |
| 6.2 | 429 | 4-7 | O43707 | Alpha-actinin 4 | 5.3 | 104854 | 82 | 18 |
| 2.2 | 1690 | 4-7 | P07355 | Annexin A2 | 7.6 | 38473 | 232 | 53 |
| -3.4 | 2085 | 4-7 | P08758 | Annexin A5 | 4.9 | 35806 | 139 | 52 |
| -2.6 | 561 | 4-7 | P15311 | Ezrin (p81) | 6 | 69268 | 150 | 36 |
| FAP Only | 894 | 4-7 | P21333 | Filamin A (Alpha-filamin) | 5.7 | 280630 | 67 | 10 |
| 2.4 | 1321 | 4-7 | Q13418 | Integrin-linked protein kinase 1 | 8.3 | 51419 | 75 | 23 |
| 2.3 | 653 | 4-7 | P26038 | Moesin | 6.1 | 67689 | 200 | 45 |
| 2 | 936 | 4-7 | P26038 | Moesin | 6.1 | 67689 | 72 | 25 |
| -2.1 | 961 | 4-7 | P26038 | Moesin | 6.1 | 67689 | 62 | 20 |
| FAP Only | 222 | 4-7 | P18206 | Vinculin (Metavinculin) | 5.5 | 123668 | 125 | 22 |
| 2.1 | 510 | 4-7 | Q15942 | Zyxin (Zyxin 2) | 6.2 | 61277 | 60 | 23 |
| FAP Only | 2430 | 4-7 | P52565 | Rho GDP-dissociation inhibitor 1 | 5 | 23076 | 78 | 38 |
| FAP Only | 1124 | 4-7 | P78371 | T-complex protein 1, beta subunit | 6 | 57357 | 66 | 18 |
| 2.7 | 2207 | 5-8 | Q01995 | Transgelin | 8.9 | 22480 | 92 | 40 |

**Supplemental Data 9**. Differential expression of cytoskeleton proteins between FAP and normal fibroblast primary cultures, organized according to functional categories.
