# Supplementary material for: APC+/− alters colonic fibroblast proteome in FAP
Source: Oncotarget. 2011 Mar 15;2(3):197–208. doi: 10.18632/oncotarget.241 (PMC3195363; doi:10.18632/oncotarget.241)
Supplement: Supplementary file 10 [file oncotarget-02-197-s010.doc]

**Supplemental Data 10. Immuno fluorescence localization of RSU-1 protein in colonic fibroblasts.** A Z-section of confocal fluorescence microscopy, 60X, oil immersion, is presented. RSU1 was visualized in green and APC in red.
