# Supplementary material for: APC+/− alters colonic fibroblast proteome in FAP
Source: Oncotarget. 2011 Mar 15;2(3):197–208. doi: 10.18632/oncotarget.241 (PMC3195363; doi:10.18632/oncotarget.241)
Supplement: Supplementary file 11 [file oncotarget-02-197-s011.doc]

**Supplemental Data 11**. Examples of gel images analysis of pH 5-8 2D gels of colonic fibroblast cultures of FAP and control patients. FAP samples are: SID-317, and SID-344 (duplicate cultures). Control samples are: SID-461, and SID-471. The RSU1 protein spots are shown in concentric circles of yellow and green.
