# Supplementary material for: APC+/− alters colonic fibroblast proteome in FAP
Source: Oncotarget. 2011 Mar 15;2(3):197–208. doi: 10.18632/oncotarget.241 (PMC3195363; doi:10.18632/oncotarget.241)
Supplement: Supplementary file 12 [file oncotarget-02-197-s012.doc]

A

B

**Supplemental Data 12. Quantitation of RSU1 expression**. Densitometric scan of the four RSU1 spots in the 2D gels of FAP versus control colonic fibroblasts (Panel A) were averaged from four gels each. The averaged values are plotted in Panel B. A ratio for the fourth spot, on far left, cannot be obtained because of the low level in FAP.
