# Supplementary material for: APC+/− alters colonic fibroblast proteome in FAP
Source: Oncotarget. 2011 Mar 15;2(3):197–208. doi: 10.18632/oncotarget.241 (PMC3195363; doi:10.18632/oncotarget.241)
Supplement: Supplementary file 13 [file oncotarget-02-197-s013.doc]

**Supplemental Data 13.** Spot 2557 in pH 5-8 gels of colonic crypts was identified as DJ-1 oncogene. It is expressed 4.8 times stronger in FAP than in Controls. It is also expressed higher in FAP colonic fibroblasts than in Control colonic fibroblasts. The first 16 gels are Controls. The next 14 gels are FAP. It is not a high abundance protein. It is necessary to perform data normalization of the spot intensities of the whole gel to obtain statistically correct comparison of spot intensities of these gels.
