# Supplementary material for: APC+/− alters colonic fibroblast proteome in FAP
Source: Oncotarget. 2011 Mar 15;2(3):197–208. doi: 10.18632/oncotarget.241 (PMC3195363; doi:10.18632/oncotarget.241)
Supplement: Supplementary file 14 [file oncotarget-02-197-s014.doc]

**Supplemental Data 14.** Quantitative Real-time PCR analysis of the mRNA levels of RSU1 in colonic fibroblast cultures. Control: SID 461, 471, 507, 508, 509. FAP: SID 516, 548, 601, 602, 608.
